# Topiramate-associated acute glaucoma in a migraine patient receiving concomitant citalopram therapy: a case-report

**DOI:** 10.1186/1757-1626-2-87

**Published:** 2009-01-26

**Authors:** Luca Spaccapelo, Silvia Leschiutta, Claudio Aurea, Anna Ferrari

**Affiliations:** 1 Headache Centre, Division of Toxicology and Clinical Pharmacology, University of Modena and Reggio Emilia, Modena, Italy; 2 Postgraduate school of Pharmacology, University of Modena and Reggio Emilia, Modena, Italy

## Abstract

We describe the case of a 34 year-old man with diagnosis of migraine with and without aura that developed myopia and acute glaucoma after 7 days of treatment with topiramate. The patient had also been taking citalopram daily for two months. Both topiramate and citalopram have been related to the increase of intraocular pressure and the development of glaucoma. We can't exclude that in this patient citalopram caused an increase of the ocular pressure in dose-dependent manner, facilitating topiramate-induced glaucoma. We recommend to pay particular attention in prescribing of topiramate in migraine patients who are already under treatment with citalopram or other antidepressants with a similar mechanisms of action.

## Introduction

Topiramate is approved as first or second choice in antiepileptic therapy, as an additional therapy for the syndrome of Lennox-Gastaut, and for the prophylaxis of migraine with or without aura in adult patients that have not responded or are intolerant to the other standard therapies [1]. Topiramate has also been prescribed for a variety of "off-label" conditions, such as affective disorders, postherpetic neuralgias, peripheral neuropathies, post-traumatic stress disorder, loss of weight, and insomnia [2].

The most common adverse events related to topiramate are represented by fatigue, paresthesia, alterations of taste, loss of weight, and drowsiness. In patients treated with topiramate, a rare but severe adverse event is represented by acute myopia with secondary closed-angle glaucoma.

In the reported cases of glaucoma, the dose of topiramate varied from 50 mgs or less (47% of cases), to 50–75 mg (33% of cases), and 100 mg (13%) to more than 100 mg (7%) and this adverse event already manifested in the 85% of cases in the first 2 weeks of treatment or at the increase of the dose [3].

Also selective inhibitors of the reuptake of serotonin (SSRI) have been related to angle-closed glaucoma [4], even if less frequently than tricyclic antidepressants. Their agonists activity at noradrenergic receptors (when present), together with passive mydriasis induced by the increase of serotonin levels, could favour the closing of the irido-corneal angle. Two cases of glaucoma have recently been reported in patients treated with citalopram, the most selective among SSRIs in the reuptake of serotonin [5,6].

We describe here the case of a patient in treatment with citalopram that developed myopia and acute glaucoma 7 days after the beginning of the therapy with topiramate.

## Case presentation

A 34 year-old man with diagnosis of migraine with and without aura, particularly worsened both for duration and for intensity in the last 3 years, was under treatment at our Headache Centre of the University Hospital of Modena. We prescribed topiramate 25 mg/day as treatment of prophylaxis. According to the prescription, the patient would have had to increase the dosing of the drug of 25 mg/day every 15 days, up to the dose of 100 mg/day, and then to return then to our centre for a checkup.

The patient had also been taking citalopram 20 mg (1 tablet/day) for two months for the treatment of an anxious-depressive syndrome. Neurological examination resulted in the normality, with symmetrical deep tendon reflexes, uninjured sensibility, absent nystagmus, and well performed vestibulo-ocular reflexes. The fundus oculi was in the limits for age, with preserved visual field. A magnetic resonance imaging (MRI), performed one month before to exclude possible organic causes of the worsening of headache, didn't detect any pathological alteration.

Seven days after the start of the treatment with topiramate, the patient complained of blurred vision and ocular pain as an intense compression. The trouble persisted during the evening, until the following morning, of unchanged intensity. For these symptoms, the patient applied to a nearby oculistic clinic, where a pressure of 40 mmHg in both eyes was found, compatible with diagnosis of acute glaucoma. The patient also presented a reduction of depth of the anterior chamber, hyperaemia of the sclera and light corneal edema. The fundus oculi showed however a rosy, not excoriated pupilla, with clean borders. The opthalmologist confirmed a severe acute myopia (right eye = -5,5 diopters, left eye = -5 diopters).

The therapy with topiramate was immediately stopped and the patient started the following antiglaucoma therapy: acetazolamide 250 mg for twice a day in tablets, latanoprost, timolol 0,50% 1 drop 3 times/day and pilocarpine 2% 1 drop for 3 times/day in eyewash. The same day, a blood sample was taken for the determination of topiramate levels by means of Fluorescence Polarized Immuno Assay (FPIA). The plasma concentration was 1,7 μg/ml, therefore below the therapeutic range (2–25 μg/ml).

Already 2 days later, ocular pressure was reduced to 14 mmHg in the right eye and to 17 mmHg in the left eye at the ophthalmological checkup. After 4 days, the depth of the anterior ocular chamber returned to normal and ocular tone subsequently reduced to 10 mmHgs in both eyes, while an important loss of the visus remained (right eye = -10,0 diopters, left eye = -6,5 diopters). The complete resolution of the symptomatology was atteined after 8 days of antiglaucoma therapy and from the suspension of topiramate: the patient eventually had a visus equal to 10/10 bilaterally, and fundus oculi and ocular pressure within the normal limits.

## Discussion

The first clinical symptoms of glaucoma (blurred vision and ocular pain) seem to be caused by an uveal effusion and by an anterior rotation of the ciliary body. The consequent ocular configuration would shallow the anterior chamber and would close the irido-corneal angle, eventually precipitating the glaucomatous crisis [7].

The molecular mechanism of this serious side effect is not clear yet, but the most shared hypothesis currently remains the idiosyncratic reaction. It seems indeed difficult to relate the mechanisms of action of topiramate (specifically within the central nervous system) to the increase of ocular pressure and the onset of angle closed glaucoma. Furthermore, it is reported that topiramate modulates voltage-dependent Na^+ ^channels, thus reducing the firing of cerebellar granule cells, and it seems contemporarily to strengthen the activity of GABA (increasing post-synaptic currents of GABA_A _receptors); finally, it would reduce the effects of the activation of glutamatergic of AMPA-kainate [8] subtype.

Topiramate is also a weak inhibitor of carbonic anhydrase (particularly of subtypes II and IV), like other sulphonamides, and acetazolamide, a drug of the same class and specific inhibitor of carbonic anhydrase, is paradoxically an effective antiglaucoma drug, also used by our patient for its activity of reduction of aqueous humor production. In particular, it is hypothesized that the antiglaucomatous activity of the sulphonamides depends on the inhibition of the isoenzyme 12 (hCA XII) of the carbonic anhydrase. Topiramate presents good affinity for this isoenzyme (KIs of 3.8 NM), while it does not show significant selectivity[9]: this observation would confirm a possible antiglaucoma mechanism of topiramate under normal conditions. Furthermore, an overexpression of the gene that codifies for this isoenzyme (hCA XII) of carbonic anhydrase has been observed in glaucomatous patients [10].

The ability to cause glaucoma seems therefore independent of the pharmacodynamic well-known effect of topiramate; the individual characteristics of the patient can probably have had a decisive role.

A peculiar element of this case is that our patient assumed daily citalopram, a selective serotonin reuptake inhibitor, as antidepressive therapy. Indeed, this drug is also implicated in the development of angle-closed glaucoma [11].

Serotoninergic receptors have been identified in the ciliary body in both animals and humans [12], suggesting that serotonin has an important role of modulation of ocular pressure. In experimental models, it has been observed that serotonin, binding to serotoninergic intraocular receptors 5-HT2 and 5-HT3, facilitates the contraction of bovine ciliary muscle, with diminution of ocular pressure [13]; but topical application of serotonin increases intraocular pressure in dose-dependent manner in the rabbit [14]. The overall effect of an increase of serotonin on intraocular pressure is not completely clarified yet. A case of acute glaucoma has been reported in a patient after an overdose of citalopram, similarly to that observed with fluoxetine [15].

On the basis of experimental and clinical data, we can hypothesize that the mechanism of action of citalopram contributes to the increasing of intraocular pressure in dose-dependent manner. Remarkable clinical consequences have not however been reported, because of changes in plasmatic levels of citalopram, caused by concomitant assumption of other drugs [16]. It is therefore unlikely that glaucoma was caused in our patient by an increase of plasmatic concentrations of citalopram, caused by topiramate-related inhibition of its elimination.

Topiramate is poorly metabolized by liver and it is a weak inducer, not a pharmaco-metabolic inhibitor. The only meaningful pharmacodynamic interaction from the clinical point of view seems to be the reduction of plasmatic concentration of estrogens in oral contraceptives, with reduction of their efficacy [17]. About 85% of unmetabolized topiramate is finally eliminated by urine; more than 16 hours after the start of symptomatology, plasmatic levels of topiramate were even lower than therapeutic range, excluding a possible dose-dependent adverse reaction to this drug.

## Conclusion

In conclusion, topiramate and citalopram were connected both with the increasing of intraocular pressure and the development of glaucoma. So far, no evidences have emerged showing that a contemporary assumption of both these drugs within therapeutic doses can increase the risk of acute glaucoma, but it has been hypothesized that selective serotonin reuptake inhibitors are able to strengthen the uveal effusion of topiramate [18].

We cannot exclude that citalopram increases ocular pressure in dose-dependent manner, through intraocular accumulation of serotonin, consequently increasing the risk of topiramate-induced glaucoma in a patient with a predisposed genetic substratum.

We therefore recommend to pay particular attention to the prescription of topiramate in patients in therapy with citalopram or other antidepressants with similar mechanisms of action.

## Consent

Written informed consent was obtained from the patient for publication of this case report. A copy of the written consent is available for review by the Editor-in-Chief of this journal

## Competing interests

The authors declare that they have no competing interests.

## Authors' contributions

LS drafted this manuscript and analized the patient data, SL and CA reviewed the current literature and helped to draft the manuscript, AF critically revised the manuscript. All authors read and approved the final manuscript.
